# Lung cancer with chest wall invasion: retrospective analysis comparing en-bloc resection and ‘resection in bird cage’

**DOI:** 10.1186/1749-8090-9-57

**Published:** 2014-03-22

**Authors:** Heron Teixeira Andrade Santos, Agnaldo José Lopes, Cláudio Higa, Rodolfo Acatauassú Nunes, Eduardo Haruo Saito

**Affiliations:** 1 Postgraduate Programme in Medical Sciences, State University of Rio de Janeiro, Boulevard 28 de Setembro, 77, Vila Isabel, 20551-030 Rio de Janeiro, Brazil

**Keywords:** Chest wall, En bloc resection, Lung neoplasm

## Abstract

**Background:**

Invasion of the chest wall *per se* is not a contraindication for tumor resection in non-small cell lung cancer (NSCLC), provided there is no mediastinal lymph node or vital structure involvement. Although widely known to Brazilian surgeons, the ‘resection in bird cage’ technique has never been widely studied in terms of patient survival. Thus, the objective of this study was to evaluate the postoperative consequences and overall survival of extra-musculoperiosteal resection compared with en-bloc resection in NSCLC patients with invasion of the endothoracic fascia.

**Methods:**

Between January 1990 and December 2009, 33 NSCLC patients with invasion of the thoracic wall who underwent pulmonary resection were retrospectively analyzed. Of the 33 patients evaluated, 20 patients underwent en-bloc resection and 13 underwent ‘resection in bird cage.’ For each patient, a retrospective case note review was made.

**Results:**

The median age at surgery, gender, indication, rate of comorbidities, tumor size and the degree of uptake in the costal margin were similar for both groups. The rate of postoperative complications and the duration of hospitalization did not differ between the groups. Regarding the outcome variables, the disease-free interval, rate of local recurrence, metastasis-free time after surgery, overall mortality rate, mortality rate related to metastatic disease, duration following surgery in which deaths occurred, and overall survival were also similar between groups. The cumulative survival curves between the ‘resection in bird cage’ and en-bloc resection and between stages Ia + Ib and IIb + IIIa + IV were not significantly different (p = 0.68 and p = 0.64, respectively). The cumulative metastasis-free survival curves were not significantly different between the two types of surgery (p = 0.38).

**Conclusions:**

In NSCLC patients with invasion of the endothoracic fascia, ‘resection in bird cage’ is a less aggressive procedure that yields similar results in terms of morbidity and mortality compared with en-bloc resection. Thus, ‘resection in bird cage’ meets the oncologic principles of resection and does not adversely affect the patients in terms of cure.

## Background

Lung cancer is an extremely complex disease in terms of its epidemiology, treatment, and prognosis. Neoplasms have the highest mortality rates in both men and women. According to the International Agency for Research on Cancer (IARC) GLOBOCAN World Cancer Report, lung cancer affects more than 1 million people a year worldwide [1]. Data from the National Cancer Institute in Brazil estimated that there were 27,320 new cases in 2012, of which 17,210 were in men and 10,110 in women [2]. Lung cancer can be classified in several ways, the most frequent of which divides the cancers into two major groups: non-small cell lung cancer (NSCLC) and small cell lung cancer (SCLC). NSCLCs have a formal indication for surgical treatment, and the preoperative evaluation should therefore be precise, seeking to recommend the best treatment approach because patient survival is closely linked to his/her tumor stage [3,4].

For adequate surgical treatment of NSCLC, en bloc resection of the affected structures is necessary, assuming that these structures are not vital structures such as the heart, large blood vessels, esophagus and trachea. The resection of these tumors is designed to include lung, parietal pleura, visceral pleura, and parts of the chest wall, which could include the rib cage, parts of vertebral bodies and the sternum [5,6]. If there is no mediastinal lymph node or vital structure involvement, chest wall invasion does not contraindicate tumor resection because lobectomy/pneumonectomy associated with thoracoplastic surgery is considered ideal for the treatment of tumors classified as T3N0-1 M0 [7]. This procedure, however, has been associated with significant morbidity and can lead to thoracic deformity, decreased pulmonary function, and postoperative pain depending on the extent of the rib resection.

The difficulty in defining the limits of chest wall invasion in IIb and IIIa stage tumors has resulted in extrapleural resections being performed only in selected cases whose results often conflict in the literature. In a retrospective study, Träsket et al. in 1984 [8] reported an actuarial survival of 75% and 28% at 5 years for en-bloc resection and extrapleural surgery, respectively. Ricci et al. in 1987 [9] also reported similar survival data. Moreover, Matsuoka et al. in 2004 [10] reported similar survival rates both in patients undergoing extrapleural resection and en-bloc resection, assuming that the resection margins were disease free. Similar findings were reported by Elia et al. [11], who also found no significant differences in the survival rates of N0 and N1 stage patients.

One alternative to radical surgery is an extra-musculoperiosteal resection, also called ‘resection in bird cage’ by its biggest advocate and creator, the Brazilian surgeon Antonio Ribeiro-Netto [12]. The surgery is characterized by extra-musculoperiosteal rib detachment, which facilitates the resection of tumors that are invading the costal face of the chest wall. In preserving the name ‘resection in bird cage’, the author pays tribute to the surgeons who used total periostomy while conserving the ribs in some forms of collapse therapy for pulmonary tuberculosis in the late eighteenth and early nineteenth century. He advocated for this technique because he believed it was less invasive, had fewer postoperative complications, and maintained the oncological principle of resection.

Although widely known by Brazilian surgeons, the extra-musculoperiosteal ‘resection in bird cage’ has never been extensively examined in terms of patient survival but has been restricted to individual case studies for each operation. We hypothesized that ‘resection in bird cage,’ in addition to being a less invasive technique, would have a similar survival rate to more radical techniques and less postoperative morbidity in the treatment of invasive lung cancer of the endothoracic fascia. Thus, the main objective of this study was to evaluate the overall survival rate of extra-musculoperiosteal resection and compare it with a more traditional surgery (en-bloc resection) in patients with NSCLC with invasion of the endothoracic fascia. Secondarily, we compared the two techniques in terms of their immediate postoperative complications following surgery.

## Methods

### Patients

Between January 1990 and December 2009, 33 patients were retrospectively analyzed who had lung cancer with invasion of the thoracic wall in a portion of the endothoracic fascia but not direct invasion of the rib cage and who underwent pulmonary resection including lobectomy, pneumonectomy, bilobectomy, and segmentectomy.

In all patients, the initial proposal was en-bloc resection. If there were visible invasion of bone or difficulty in mobilizing the tumor through the chest wall, the en-bloc resection was immediately performed to preserve the oncological principle of resection. Despite the contribution given by imaging methods performed preoperatively, the indication for ‘resection in bird cage’ was done only during the surgery if local conditions allow the procedure. When evaluating the resection of the rib cage, the surgeon identified that there was no macroscopic invasion of the bone and the tumor invaded only endothoracic fascia. This was confirmed by analysis of frozen tissue samples (periosteum, endothoracic fascia and parietal pleura) performed during the surgery.

Of the 33 patients evaluated, 20 patients underwent en-bloc resection, and 13 underwent ‘resection in bird cage.’ For each patient, a retrospective case note review that included the following variables was made: age at surgery, gender, tobacco use history and the presence of chest pain and comorbidities. The following variables were also analyzed: tumor location, histological type, operative mortality, length of hospital stay, postoperative tumor stage, disease-free interval, local disease recurrence, and overall survival. The study was approved by the Research Ethics Committee of State University of Rio de Janeiro.

### Surgical procedures

**Figure 1:**
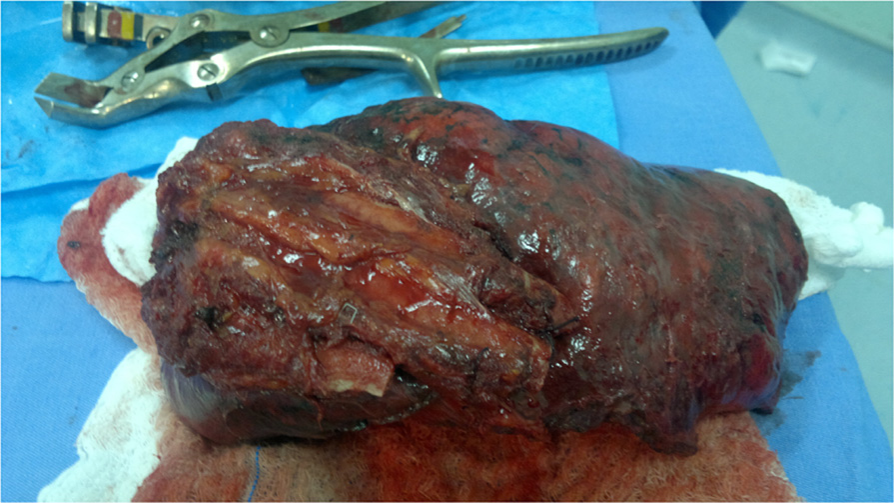
Lobectomy with en-bloc resection.

Radical surgery was performed via en bloc resection of the pleural surfaces together with the endothoracic fascia, ribs, and intercostal muscles, which at times extended to more superficial tissues (muscles and fat tissue). In general, the tumor was removed with a safety margin varying with local surgical conditions. Pneumonectomy was performed whenever the mass crossed over the major fissure. Sometimes the surgery involved resection of multiple ribs that were in close contact with the tumor. Depending on the extent of the resection, chest wall reconstruction was required using special screens (Marlex or polypropylene) or bone cement (methyl methacrylate) [5,6]. This reconstruction was done in order to reduce the risk of respiratory instability by loss of part of the rib cage (Figure 1).

**Figure 2:**
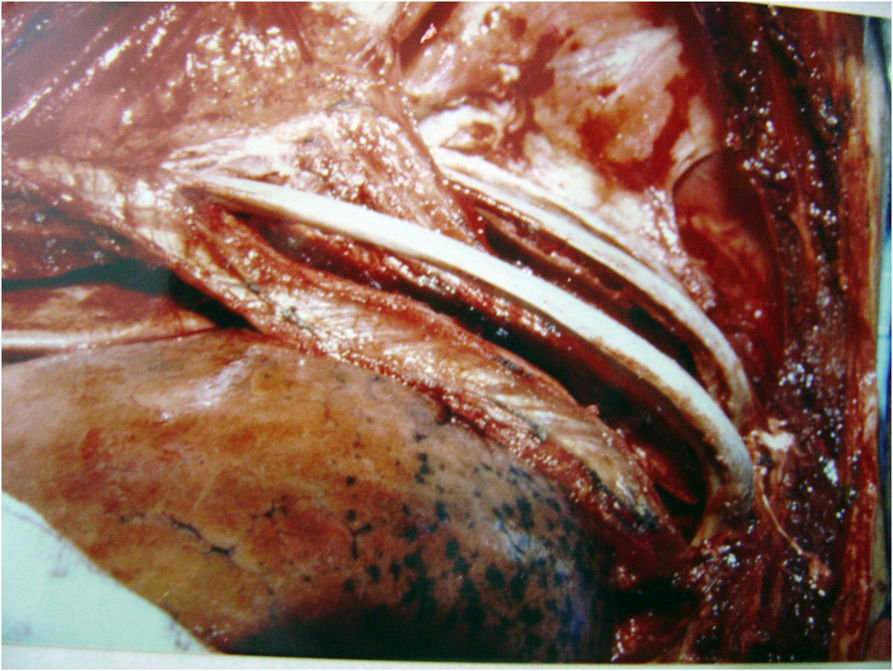
Depiction of the ‘resection in bird cage’ technique with soft tissue adhered to the tumor.

The ‘resection in bird cage’ [12] consisted of removal of the lung (bronchopulmonary segment, lung lobe or an entire lung) together with structures that were attached to the tumor. The procedure characterized by extra-musculoperiosteal rib detachment. The surgery involved the removal of the periosteum with the parietal pleura and endothoracic fascia, intercostal muscles and neurovascular bundle at an interval of two intercostal spaces above and below the rib region affected. The anterior-posterior extent of resection was generally 5 cm before and after the affected area. In some cases, this technique was extended from in front of or behind the plane of the mammary vessels until the spinal column (Figure 2).

### Statistical analysis

To check the homogeneity of the sample, the Shapiro-Wilk test was used; if a meaningful number of variables did not have a normal distribution, then nonparametric tests were selected. The results were expressed as the median (minimum and maximum values) or number (percentage). Numerical variables were compared using the Mann–Whitney test. Categorical variables were compared using the chi-squared test and Fisher’s exact test. The cumulative survival and cumulative metastasis-free survival curves were adjusted using the Kaplan-Meier method. Log rank statistics were used to determine whether there were any significant differences in the curves stratified by type of surgery and postoperative tumor stage. Data analysis was performed using SAS 6.11 software (SAS Institute, Inc., Cary, NC, USA). The statistical significance level was set at p < 0.05.

## Results

Of the 33 patients studied, 23 were men with a median age of 62 years at surgery (range, 39 to 77 years). Twenty patients had chest pain, whereas 15 patients reported comorbidities, the most frequent of which included systemic hypertension (9 cases), chronic obstructive pulmonary disease (8 cases), diabetes mellitus (4 cases), and dyslipidemia (3 cases). A smoking history of >40 pack-years was reported by 21 patients. A forced expiratory volume in 1 second (FEV_1_) of >1.5 liters was observed by 23 patients. The tumor locations had the following distribution: right upper lobe (15), left upper lobe (10), left lower lobe (4), right lower lobe (2), right upper lobe and middle lobe (1), and left upper lobe and bottom lobe (1). The tumor was >3 cm in 26 patients. Bone scans showed uptake in the costal arch in 5 cases. The preoperative tumor stage was documented for 29 patients and included the following distribution: stage Ib (2), stage IIb (21), stage IIIa (5), and stage IV (1).

The postoperative tumor stage was documented in 26 patients and included the following distribution: stage Ia (1), stage Ib (8), stage IIb (11), stage IIIa (5), and stage IV (1). The histological tumor types included both adenocarcinoma and squamous carcinoma, which were present in 11 patients. There was one case of operative mortality (pneumonia), and morbidity was reported 19 cases, the primary cause of which was pulmonary infection (9 patients). The median duration of hospitalization was 40 days (range, 10 to 177 days). Postoperatively, 9 patients underwent radiotherapy, and 4 patients underwent chemotherapy.

The median disease-free interval was 28.5 months (range, 15 days to 186 months). Local disease recurrence was documented in 2 cases. For the locations of the 13 cases of metastasis, an analysis of medical records indicated that the brain was the organ most often affected (6 cases) followed by the bone (3 cases). The median metastasis-free time following surgery was 12 months (range, 1 to 55 months). The median overall survival was 28 months (range, 15 days to 186 months). There were 14 documented deaths, and the median survival time following surgery was 12 months (range, 15 days to 98 months). Ten of the deaths were related to metastatic disease.

Table 1, Table 2 and Table 3 compare the clinical characteristics, lung function, parameters related to surgery, and outcome variables between en-bloc resection and ‘resection in bird cage.’ The preoperative tumor stage was determined in 17 patients undergoing en-bloc resection and included the following distribution: stage IIb (13) and stage IIIa (4). The preoperative oncologic staging was determined in 12 patients undergoing extra-musculoperiosteal resection ‘in bird cage’ and included the following distribution: stage Ib (2), stage IIb (8), stage IIIa (1), and stage IV (1). The postoperative tumor stage was determined in 16 patients undergoing en-bloc resection and included the following distribution: stage IIb (11) and stage IIIa (5). The postoperative tumor stage was determined in 10 patients undergoing ‘resection in bird cage’ and included the following distribution: stage Ia (1), stage Ib (8), and stage IV (1).

**Table 1:** Clinical data and lung function of patients who underwent en-bloc resection and ‘resection in bird cage’

	**En-bloc resection**		**‘Resection in bird cage’**		
	**N**	**Value**	**N**	**Value**	**p *- *** **value**
Age at surgery (years)	20	64 (24–77)	13	61.5 (39–75)	0.62
Gender male	20	13 (65%)	13	10 (76.9%)	0.37
Chest pain	20	15 (75%)	13	5 (38.5%)	**0.036**
Comorbidities	20	11 (55%)	11	4 (36.4%)	0.32
Systemic hypertension		6 (30%)		3 (27.3%)	0.66
Chronic obstructive pulmonary disease		5 (25%)		3 (27.3%)	0.80
Diabetes mellitus		3 (15%)		1 (9.10%)	0.72
Dyslipidemia		2 (10%)		1 (9.10%)	0.81
Lung function	18		11		
FEV_1_ > 1.5 L		16 (88.9%)		10 (99.9%)	0.76
FEV_1_ (% predicted)		66.5 (54–78.3)		72 (57–85)	0.51
FVC (% predicted)		81 (67–88)		85 (70–92.5)	0.67
FEV_1_/FVC (%)		73 (58–84)		74 (60–87)	0.72
Tumor >3 cm	19	19 (100%)	9	7 (77.8)	0.58
Bone scintigraphy uptake in costal arch	8	4 (50%)	8	1 (12.5%)	0.21

Results expressed as median (minimum and maximum values) or number (percentage). N = Represents the total number of cases in which the finding was evaluated retrospectively. *FEV*_*1*_ Forced expiratory volume in 1 second, *FVC* Forced vital capacity.

**Table 2:** Parameters related to the surgical procedure

	**En-bloc resection**		**‘Resection in bird cage’**		
	**N**	**Value**	**N**	**Value**	**p-value**
Postoperative complications	17	10 (58.8%)	13	9 (69.2%)	0.42
Duration of hospital stays (days)	14	36.5 (10–62)	11	55 (25–177)	0.13
Postoperative radiotherapy	18	8 (44.4%)	11	1 (9.10%)	0.052
Postoperative chemotherapy	17	3 (17.7%)	10	1 (10%)	0.91

Results expressed as median (minimum and maximum values) or number (percentage). N = Represents the total number of cases in which the finding was evaluated retrospectively.

**Table 3:** Outcome variables of patients who underwent en-bloc resection and ‘resection in bird cage’

	**En-bloc resection**		**‘Resection in bird cage’**		
	**N**	**Value**	**N**	**Value**	**p-value**
Disease-free interval (months)	14	28.5 (0.5–175)	10	23 (1–186)	0.61
Local recurrence	15	1 (6.7%)	12	1 (8.3%)	0.94
Detection of metastases	14	6 (42.9%)	11	7 (63.6%)	0.26
Metastasis-free time after surgery (months)	6	12 (2–55)	5	7 (1–12)	0.075
Deaths	16	9 (56.3%)	12	5 (41.7%)	0.35
Deaths related to metastatic disease	13	6 (46.2%)	8	4 (50%)	0.60
Duration in which deaths occurred after surgery (months)	8	14 (0.5–64)	3	9 (2–98)	0.91
Overall survival (months)	15	30 (0.5–175)	12	26 (2–186)	0.71

Results expressed as median (minimum and maximum values) or number (percentage). N = Represents the total number of cases in which the finding was evaluated retrospectively.

**Figure 3:**
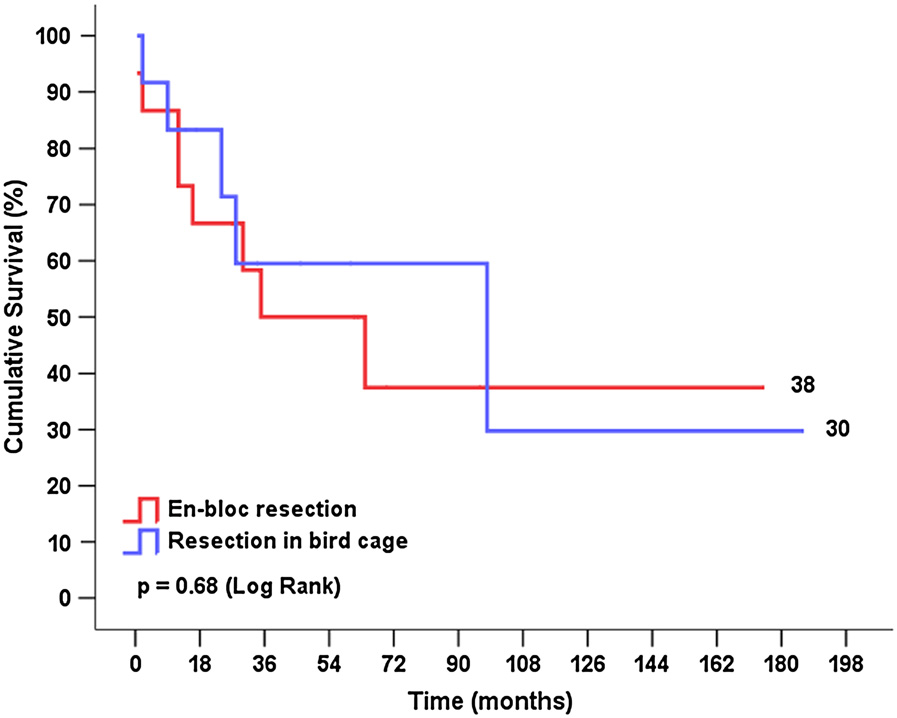
Survival curve according to the type of surgery.

**Figure 4:**
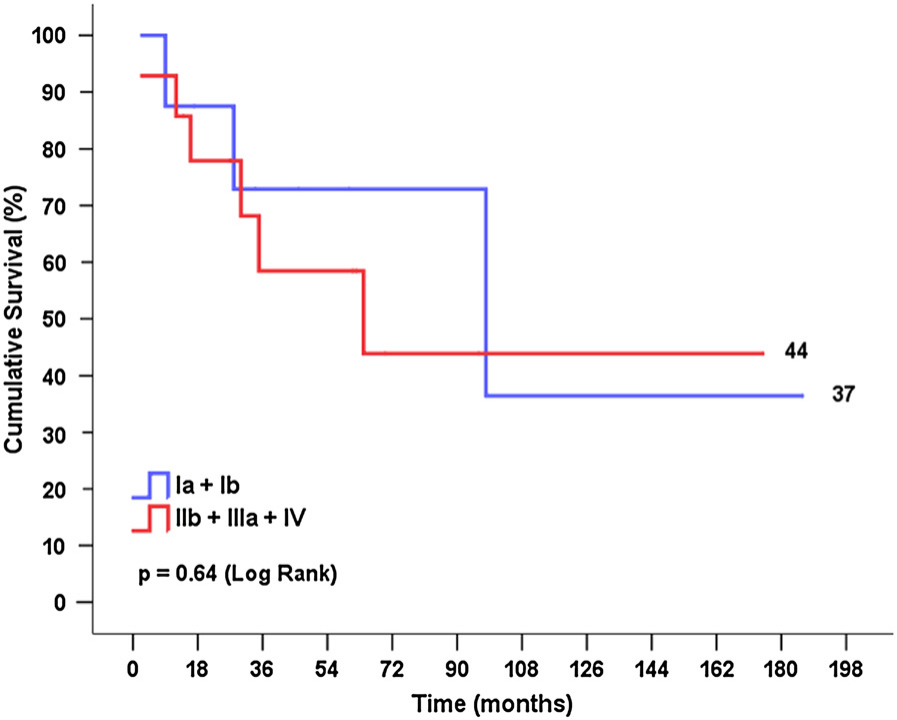
Survival curve according to the tumor stage postoperatively.

**Figure 5:**
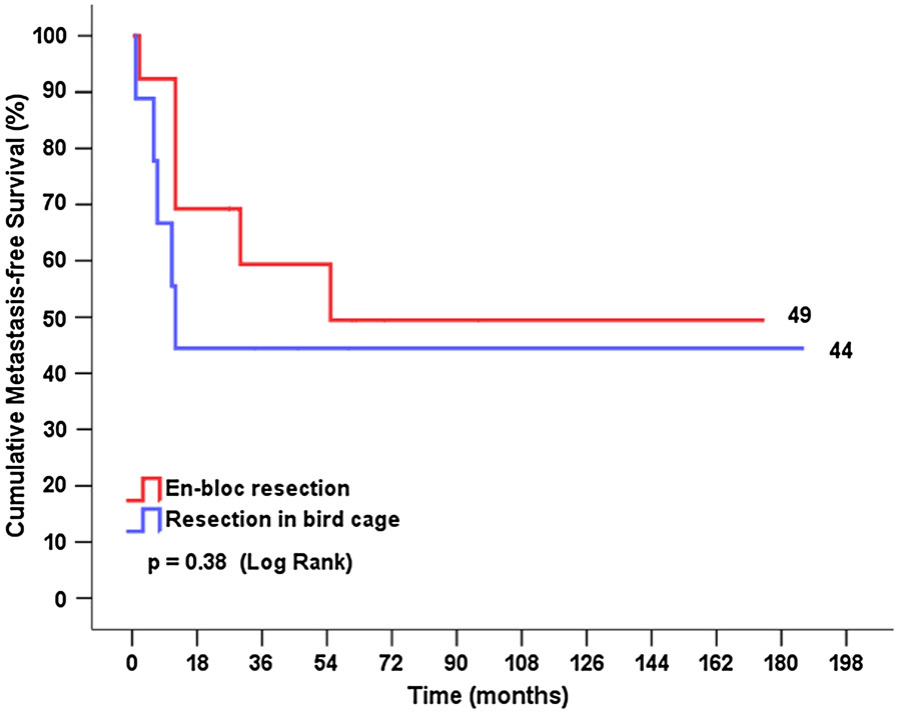
Metastasis-free survival curve according to the tumor stage postoperatively.

Figures 3 and 4 show the cumulative survival stratified by the type of surgery and the postoperative tumor stage, respectively. The curves were compared using log rank statistics, which indicated that there were no significant differences between cumulative survival rates for ‘resection in bird cage’ and en-bloc resection or between stages Ia + Ib and IIb + IIIa + IV (p = 0.68 and p = 0.64, respectively). Figure 5 shows the cumulative metastasis-free survival, which was also not significantly different between the two types of surgery (p = 0.38).

## Discussion

The main finding of this study was that extra-musculoperiosteal ‘resection in bird cage’ and en-bloc resection produced similar results in terms of both mortality and survival in NSCLC patients with invasion of the endothoracic fascia. Moreover, we found that the rate of postoperative complications and length of hospitalization were not significantly different between the two techniques. To the best of our knowledge, this is the first study to compare en-bloc resection and ‘resection in bird cage’ in both the short- and long-term.

In the present study, the median age at surgery was 62 years, and the predominant gender was male (69.7% of cases), which are data consistent with other studies [6,13]. Our patient sample pool also indicated that there were similar numbers of adenocarcinoma and squamous cell carcinoma histological types (11 cases each). Although it is known that adenocarcinoma is the most common histological tumor type found in large centers in the U.S. and Europe [14,15], squamous cell carcinoma is still the most common type in Brazil despite the gradual narrowing of the difference in frequency between them in recent years [16,17].

Because the present study aimed to evaluate the peripheral tumors that come into contact with the chest wall, chest pain was expected to be the predominant symptom. In fact, the most prominent symptom for most patients (60.6%) was pain in the rib cage. Stoelben and Ludwig [18] evaluated several studies involving chest wall resection in NSCLC and reported rates of chest pain between 39% and 60%. Our patient sample had a higher rate of chest pain, which suggested that our patients had a higher level of rib cage involvement at diagnosis.

The difficulty in identifying whether there is invasion of the costal margin before surgery has been an issue for a long time. Although chest pain is the main complaint of a large number of patients, it presence *per se* is not pathognomonic of chest wall involvement. The use of imaging methods such as computed tomography, ultrasound, magnetic resonance imaging, positron-emission tomography and bone scintigraphy increases the chance of success, although the difference between T2 and T3 tumors can be less than 1 mm and occupy just a small amount of surface area, hindering both the surgical and histological analyses [18,19]. In the present study, uptake into the costal margins adjacent to the tumor occurred in 5 out of the 16 patients who were imaged using bone scintigraphy, which corroborated our suspicion for costal margin invasion. Interestingly, several publications have shown that the degree of invasion does not appear to impact survival if there is a complete resection [20,21]. However, some studies [13,22] have reported a better prognosis for patients with tumor invasion of the parietal pleura compared with patients who have deep invasion.

In the present study, the analysis of overall survival for the entire sample population revealed that the median survival time was 28 months. This number differs from the data reported by Chapelier et al. in 2000 and Lee et al. in 2012, who observed a median survival time of 15.9 and 18 months, respectively [13,22]. However, Suzuki et al. [23] reported a mean follow-up time of 49.3 months (range, 1 to 207 months). More recently, Kawaguchi et al. [6] evaluated 11,663 patients using the Japanese Joint Committee of Lung Cancer Registry and selected 531 patients with tumors invading the chest wall who underwent surgery. These authors reported a median survival time of 46 months. The data are conflicting because many other factors may be involved in the prognosis, including lymph node status, tumor size, tumor cell biology, and the degree of local invasion [13,23-25]. Moreover, many studies take into consideration only complete resection cases; thus, a comparative analysis with our data may not be pertinent.

Surgical management of NSCLC invasion of the endothoracic fascia is still a controversial issue in the literature [10,11,21,25]. Our study compared the most frequently used surgical technique for this type of neoplasm (en-bloc resection) to another technique that has not yet been reported in the literature (‘resection in bird cage’) although it is widely used by surgeons in Brazil. The ‘resection in bird cage’ is performed only in those patients with NSCLC that are in contact with the chest wall, but that is not identified gross invasion of the rib cage. The invasion is acceptable only up to endothoracic fascia by analysis of frozen tissue samples. If the surgeon identifies the invasion macroscopically, the technique to be adopted is the en-bloc resection which is the standard technique [5,6]. The ‘resection in bird cage’ is a less aggressive technique and avoids en-bloc resection sometimes unnecessary [12]. A recent publication [13] mentions an extended extrapleural resection for deep invasion of the chest wall but does not offer specific details on this type of approach, making it impossible to compare it with our extra-musculoperiosteal ‘resection in bird cage.’ However, several studies have compared en-bloc resection to pleurectomy, reporting conflicting results. Whereas Albertucci et al. [26] and Trastek et al. [8] reported improved survival with en-bloc resection compared with pleurectomy (even in the absence of extra-pleural complications), Downey et al. [27] reported that extra-pleural resection without tumor violation had a higher survival rate.

Despite a higher cumulative survival rate at 5 years with ‘resection in bird cage’ (60% vs. 38%, p < 0.001) (Figure 3), we observed that the cumulative survival rates for en-bloc resection and ‘resection in bird cage’ were similar to the results at the end of the follow-up for this cohort (38% and 30%, respectively, p = 0.68). However, the cumulative survival rates at 5 years reported by Lee et al. [13] and Kawaguchi et al. [6] for cohorts who underwent the different surgical techniques were 26.3% and 44.9%, respectively. Several factors have an impact on the survival of NSCLC patients, of which mediastinal lymphadenopathy is regarded as one of the most important [3,28]. Thus, the new TNM classification (7th edition) takes into consideration the lymph node status when raising the tumor stage from IIb to IIIa by accounting for metastases in the lymph nodes (N1 or N2) [3,4]. Even raising tumors that are >7 cm to T3 does not interfere with the ultimate tumor stage, although additional analyses that address this issue are recommended. Some authors suggest that tumor size *per se* has an important impact on prognosis [5,6]. Another important observation noted in our study was the low number of local recurrences, suggesting that the oncological treatment principle of complete resection was achieved with both techniques. It is noteworthy that more patients underwent radiotherapy in “en bloc” group possibly because these patients had a more advanced postoperative tumor stage. Although the cumulative metastasis-free survival curve slightly favored the en-bloc resection technique, we did not observed any significant differences compared with the ‘resection in bird cage’ technique (p = 0.38).

A critical analysis of these results and their limitations is very important. This study was performed using only a small number of patients via a retrospective method to review the data. Moreover, the missing data impacted on differences in the sample size for each patient characteristic and outcome measures, which may have influenced the results. Thus, there is need for intervention studies with proper design and larger sample aiming at confirm these preliminary results. However, because this is the first study to report the long-term results of the surgical technique known as ‘resection in bird cage,’ we believe our results provide an important contribution to the field.

## Conclusions

In NSCLC patients with invasion of the endothoracic fascia, extra-musculoperiosteal ‘resection in bird cage’ is a less aggressive procedure that yields similar results in terms of morbidity and mortality compared with en-bloc resection. Furthermore, the overall survival rate, cumulative survival rate and cumulative metastasis-free survival rate were comparable between the two techniques, suggesting that the ‘resection in bird cage’ technique achieves the oncologic principles of resection without adversely affecting patients in terms of cure.

## Abbreviations

FEV1: Forced expiratory volume in 1 second; FVC: Forced vital capacity; IARC: International agency for research on cancer; NSCLC: Non-small cell lung cancer; SCLC: Small cell lung cancer.

## Competing interests

The authors declare that have no competing interests.

## Authors’ contributions

HTAS and AJL conducted literature reviews, and drafted the manuscript. HTAS, AJL, CH, RAN, and EHS designed the study and helped to draft the manuscript. HTAS, CH, RAN, and EHS carried out the study, collected the data and helped to draft the manuscript. All authors read and approved the final manuscript.
